# Next-generation mammalian genetics toward organism-level systems biology

**DOI:** 10.1038/s41540-017-0015-2

**Published:** 2017-06-05

**Authors:** Etsuo A. Susaki, Hideki Ukai, Hiroki R. Ueda

**Affiliations:** 1 Department of Systems Pharmacology, Graduate School of Medicine, The University of Tokyo, 7-3-1 Hongo, , Bunkyo-ku, Tokyo 113-0033 Japan; 2 Laboratory for Synthetic Biology, RIKEN Quantitative Biology Center, 1-3 Yamadaoka, , Suita, Osaka 565-0871 Japan; 3 PRESTO, Japan Science and Technology Agency (JST), 4-1-8 Honcho, , Kawaguchi, Saitama 332-0012 Japan

## Abstract

Organism-level systems biology in mammals aims to identify, analyze, control, and design molecular and cellular networks executing various biological functions in mammals. In particular, system-level identification and analysis of molecular and cellular networks can be accelerated by next-generation mammalian genetics. Mammalian genetics without crossing, where all production and phenotyping studies of genome-edited animals are completed within a single generation drastically reduce the time, space, and effort of conducting the systems research. Next-generation mammalian genetics is based on recent technological advancements in genome editing and developmental engineering. The process begins with introduction of double-strand breaks into genomic DNA by using site-specific endonucleases, which results in highly efficient genome editing in mammalian zygotes or embryonic stem cells. By using nuclease-mediated genome editing in zygotes, or ~100% embryonic stem cell-derived mouse technology, whole-body knock-out and knock-in mice can be produced within a single generation. These emerging technologies allow us to produce multiple knock-out or knock-in strains in high-throughput manner. In this review, we discuss the basic concepts and related technologies as well as current challenges and future opportunities for next-generation mammalian genetics in organism-level systems biology.

## Introduction

Systems Biology is a natural extension of molecular and cellular biology,^1–3^ which consists of multi-stage processes beginning with a (1) comprehensive identification and (2) quantitative analysis of individual system components and their networked interaction, which leads to the ability to (3) control existing systems toward the desired state and (4) design new systems based on an understanding of the underlying structural and dynamical principles. After identification of key genes by classical forward and reverse genetics, systems biology in mammals has been further accelerated by a series of genome projects, especially at the molecular-to-cellular levels, where in vitro cell culture systems allow system-level identification, analysis, control, and design of molecular networks. On the other hand, organism-level systems biology in mammals still remains an important challenge in biology.^4^

In order to identify and analyze molecular networks and/or cellular circuits in organisms, gene knock-out (KO) or knock-in (KI) are powerful technologies often used in mammalian reverse genetics. However, this classical genetics requires several generations of crosses to produce mutant animals of sufficient quality and quantity for phenotype analysis. The time consuming conventional methods for producing KO or KI mice usually involve targeting-vector construction (2 weeks to a few months; depending on the complexity of constructs), the introduction of target mutations into embryonic stem cells (ESCs) by homologous recombination (a few weeks), and the injection of the mutant ESCs into wild-type blastocysts to produce chimera mice (~3 weeks). If the mutant ESCs contribute to the germ-line of the newborn chimera mice, their next-generation offspring will possess a heterozygous mutation (~3 months). Further crossings of the offspring (several months to years; at least 3 months per generation) will produce mice with completely homozygous KO or KI mutations on an inbred genomic background, which is required for reliable phenotype analysis. Thus, conventional methods require substantial amounts of time, space, and effort to knock out or knock in even a single gene. Therefore, to comprehensively identify and quantitatively analyze molecular networks and/or cellular circuits in organisms in an efficient manner will require next-generation genetics, i.e., genetic alterations without crossing. In this review, we discuss the basic concepts and related technologies as well as current challenges and future opportunities for next-generation mammalian genetics in organism-level systems biology.

## Conventional mammalian genetics

Mammalian genetics (particularly in mice) has been widely exploited in order to investigate complex and dynamic biological processes executed by molecular networks and/or cellular circuits in organisms. Forward genetics (germline mutagenesis and gene-trap) and reverse genetics (targeted KO or KI) are available in mouse genetics as in other model organisms such as yeast, nematode and fly. Especially, developmental engineering based on the establishment of cultured ESCs was often used to generate KO and/or KI mice.^5–7^ Various genetic tools can be also introduced by transgenic (Tg) mice techniques.^8^

**Fig. 1:**
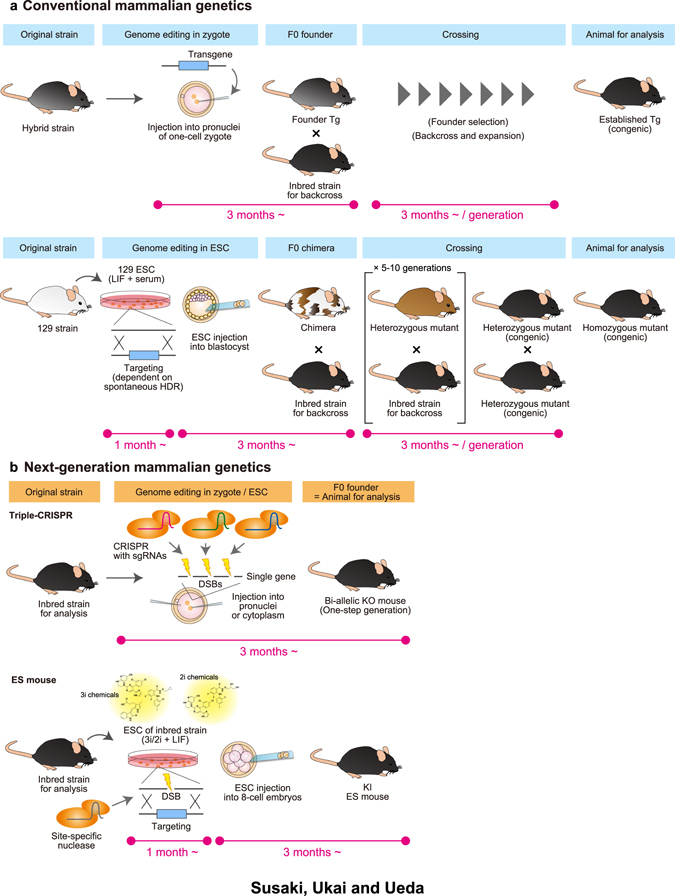
Conventional and next-generation mammalian genetics. **a** A typical procedure for conventional mouse genetics. *Upper panel*: generation of a transgenic mouse, *lower panel*: gene targeting in ESCs and generation of the mutated mouse. An inbred strain such as C57BL/6 (B6) is widely used for final analysis, while hybrid or other inbred strains are used in the production stages for practical reasons. Therefore, a prolonged backcross procedure is needed in many cases. In addition, gene targeting in ESCs is dependent on a spontaneous DSB and following HDR in the cells, causing an inefficient targeting rate. **b** In next-generation mouse genetics, all of the crossing procedures are not needed because of the use of an inbred strain for analysis, efficient genome editing in zygotes or ESCs mediated by site-specific endonucleases, and one-step generation of the genome-edited bi-allelic KO mouse or KI ES mouse. These F0 animals can be used in subsequent phenotyping experiments

However, the production of genome-edited mice has been generally low-throughput, and needed huge time and effort in the conventional ways (Fig. 1a). For example, a Tg mouse strain is produced by pronuclear injection of a DNA fragment harboring a transgene, which is randomly integrated. Therefore, non-specific expressions of the transgene are usually observed in the resultant strain and the F0 founders must be selected and further expanded for use as the strains for the subsequent research. In case of gene targeting in ESCs, a chimera mouse (mouse having both ESC and host embryo-derived cells) is first produced by injection of the ESCs into blastocyst-stage embryos. If the injected ESCs by chance contribute to germ-line cells, the resultant F0 chimera can transfer the introduced mutation to the next F1 generation. Therefore, the homozygous mutants can be obtained, in principle, at least in the third (F2) generation, which takes 9 months after ESC injection. However, these procedures are not robust and it usually takes longer because of low targeting rates in ESCs, low germ-line transmission rate in chimera, or unexpected infertility of the created mutant strain. Furthermore, because a mixed genetic background can cause phenotypical alterations which make the experimental results difficult to interpret,^9^ the generated strains additionally need to be backcrossed to a ‘standard’ inbred strain such as C57BL/6 (hereafter denoted as B6) several times. This labor-intensive step is practically required in most cases because F1 hybrid strains or 129 strain-derived ESCs are commonly used in Tg zygote production or targeting in ESCs, respectively due to their higher viability or efficient germ-line transmission in F0 chimera.

Despite the limitations of conventional mammalian genetics, systematic, large-scale mouse genetics projects have been performed. For example, ethyl-nitrosourea mutagenesis in mice was exploited to screen mammalian circadian clock genes^10–12^ and for systematic gene function studies.^13, 14^ The gene-trap strategy has more recently been applied to such forward-genetics approaches, and >100,000 of trapped ESC lines have been established and kept in international organizations (e.g., International gene trap consortium or IGTC, http://www.genetrap.org).^15^ Other systematic international efforts to collect, prepare and maintain mutant mice and ESCs have also been performed, such as the International Knockout Mouse Consortium/International Mouse Phenotype Consortium (http://www.mousephenotype.org).^16–19^ Multiple Cre Tg/KI strains have also been established by individual researchers, institutes and international consortiums.^20–22^ However, to carry out organism-level systems biology, these large-scale efforts should be scaled down to the single-laboratory scale or even to the single-researcher scale. To address this technological challenge, next-generation mammalian genetics without crossing is proposed here to allow completion of KO or KI mouse production and phenotyping analysis within the F0 generation (Fig. 1b). This can be realized by the application of highly efficient genome editing by site-specific nucleases for one-step generation of whole-body genome-edited inbred animals within a single generation. Recently, there has been rapid progress in next-generation mammalian genetics, as introduced below, which will form an essential platform for organism-level systems biology.

## Current technologies for efficient genome editing by site-specific endonucleases

**Fig. 2:**
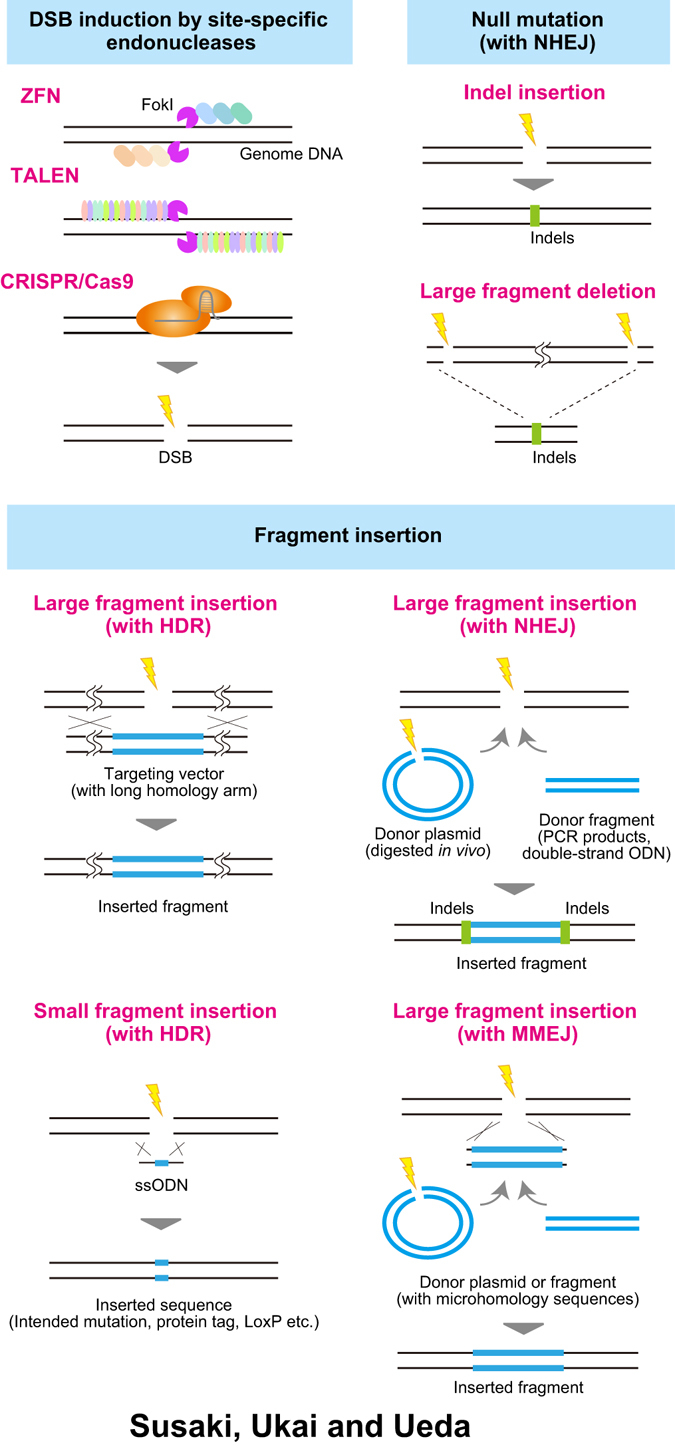
DSB-mediated genome editing. *Upper left*: type of site-specific endonucleases which are recently used for efficient genome editing purposes. *Upper right*: introduction of a null mutation by DSB. When repaired by NHEJ pathway, small deletion or insertion of nucleotides (indels) occurred at the joint site, which cause a nonsense or missense mutation in the targeted ORF. Long deletions can also be introduced by multiple DSBs. *Lower panels*: strategies of fragment insertion. Homology-directed repair (HDR) supports insertion of a large or a small fragment with homology sequences. NHEJ also supports the insertion of a large fragment without homology sequence, although inserted direction is not controllable and indels are introduced at the joint regions. Microhomology-mediated end joining (MMEJ) mediates fragment insertion with very short (10–40 bp) microhomology arms and thus potentially ameliorates drawbacks in the other two pathways

Double-strand breaks (DSBs) lead to several DNA repair pathways, such as (1) homology-directed repair (HDR) where a homologous DNA sequence is used for recombination or annealing,^23^ (2) non-homologous end joining (NHEJ), where the broken ends are directly reconnected with a frequent insertion or deletion of a random number of bases (denoted as “indels”),^24^ (3) microhomology-mediated end joining (MMEJ), where a small microhomology fragment is used for the end-joining.^25^ Induced DSB and the following repair processes enable highly efficient genome editing (both KO or KI) at the broken locus. Although the genome editing induced by DSBs was previously investigated by introduction of *I-Sce*I (a mitochondrial endonuclease from *S. cerevisiae*) into cultured mammalian cells, which stimulated extrachromosomal homologous recombination,^26^ DSB-induced genome editing has been practically used in recent years. We review such efficient genome editing methods using site-specific nucleases, which can accelerate the production of KO and KI mice via relatively simple steps and thus help realize next-generation mammalian genetics (Fig. 2).

### Site-specific endonucleases used in modern genetics

Three major classes of site-specific endonucleases have been used for genome-editing,^27^ zinc-finger nuclease (ZFN),^28^ transcription activator-like effector nuclease (TALEN)^29^ and clustered regularly interspaced short palindromic repeats (CRISPR)-associated Protein9 (Cas9).^30^ ZFN and TALEN are categorized into customizable endonucleases because they are composed of a customizable sequence-specific DNA-binding domain fused to a nonspecific DNA catalytic domain of *Fok*I endonuclease.^31^ On the other hand, Cas9 is a RNA-guided endonuclease and recruited to specific DNA sequences by a short RNA guide molecule that recognizes target DNA via base-pairing.^32^

The DNA-binding domain of ZFN contains 3–6 arrays of Cys2-His2 zinc finger motif.^33, 34^ The individual zinc finger recognizes 3 bp in the major groove of DNA.^35^ Selected zinc-finger modules that recognize nearly all of the 64 possible nucleotide triplets have been developed. Therefore, an assembled custom array of six zinc fingers can be constructed to recognize a unique 18 bp sequence.^34, 36–38^ This length of target sequence covers a possible 68 billion unique DNA sequences.

TALEN contains another type of customizable DNA-binding domain, which is composed of a tandem 33–35-amino acid repeat (TALE repeat) derived from the plant pathogenic bacteria genus *Xanthomonas*.^39, 40^ The individual TALE repeat recognizes a single base pair via two hypervariable amino acid residues inside the repeat (repeat-variable di-residues or RVDs). The four most common RVDs (HD, NG, NI, and NN) are known to recognize each of the four nucleotides (C, T, A, and G). Therefore, the tandem TALE-repeat is usually constructed with approximately 18 TALE repeats of different base pair-binding specificities, under consideration of its limitation that TALE-binding sites should start with a T base. The TALE repeat domain generally gives similar DNA-binding specificity and more flexibly when compared with ZFNs.^41^

Dimerization of the *Fok*I endonuclease catalytic domain is essential for cleavage of DNA by ZFN and TALEN.^31^ This means that two ZFN or TALEN molecules must bind on both right and left sides of the target site with an appropriate orientation and spacing. Therefore, the dimer recognizes 2-fold longer sequence at the target site than single ZFN or TALEN molecules. This molecular property gives higher specificity and reduced off-target effect.

Unlike the former molecules, Cas9 is an RNA-guided DNA endonuclease derived from the type II bacterial adaptive immune system CRISPR, and is recruited to specific target sequences by two short RNA molecules:^32^ the CRISPR RNA (crRNA) which anneals with the target sequence, and the trans-activating crRNA (tracrRNA) which is partially complementary to the crRNA and anneals to the crRNA. This two-component RNA system was further simplified to synthetic single-guide RNA (sgRNA) consisting of a fusion of crRNA and tracrRNA.^42^ The target sequence in the CRISPR/Cas9 system can be readily changed by simply re-designing a part (around 20 bp) of the crRNA or sgRNA. This simplicity is in contrast to the much more burdensome procedures in ZFN and TALEN vector construction. This simplicity endows the CRISPR/Cas9 system with a significant advantage for use as a site-specific endonuclease for various genome editing purposes, including multiple gene KO,^43, 44^ or even genome-wide gene perturbations.^45, 46^

Many studies have tried to increase the flexibility and decrease any off-target effect of the CRISPR/Cas9 system for practical use. The DNA cleavage activity of Cas9 molecules is dependent on the presence of a short (around 2–6 nucleotides) protospacer adjacent motif (PAM), which is located beside the complemental sequence of crRNA/sgRNA-targeted region.^47^ PAM sequence varies according to the CRISPR-based systems and organisms, and restricts the flexibility of the target sequence. However, the sequence dependency of the Cas9 molecule can be artificially modified and such PAM engineering can expand the target range of the system.^48, 49^ Other modification of Cas9 molecules contribute to off-target suppression. Cas9 induces DSBs at approximately three bases upstream of the PAM by two endonuclease domains, a RuvC-like endonuclease domain (RuvC domain) and a HNH-like endonuclease domain (HNH domain), which are located at the amino terminus and the mid-region of the Cas9, respectively.^50^ The RuvC domain cleaves the non-complementary strand while the HNH domain cleaves the crRNA-complementary strand. Inactivation of these endonuclease domains via point mutations can convert Cas9 endonuclease into a DNA “nickase” that creates a single-stranded break, which reduces off-target activity by 50-fold to 1500-fold in cell lines and zygotes without sacrificing on-target cleavage efficiency.^51, 52^ Others have tried *Fok*I-dCas9 fusion protein as a dimer to improve targeting specificity by their recognition of distinct sites.^53, 54^ Use of truncated sgRNA can also suppress undesired off-target activity by >5000-fold without sacrificing on-target genome editing efficiency, possibly by decreasing the sgRNA-DNA interface.^55^

### Introduction of null mutation

DSBs induced by site-specific endonucleases activate an internal DSB-repair pathway, which is exploited for efficient genome editing. Among them, NHEJ-dependent indel insertion is the simplest and the most effective method for gene KO^24, 25^ (Fig. 2). Indels in the open reading frame (ORF) of targeted gene lead to loss-of-function mutation by creating frame-shift mutations or an accidental stop codon at the cleavage site. The NHEJ-dependent gene KO works stably in mammalian cultured cells,^28, 30, 56^ ESCs and other pluripotent cells, or even mammalian zygotes.^57–59^ Furthermore, simultaneous use of multiple sgRNAs can introduce mutations in multiple genes and create a large deletion between the targeted loci, as well as increase the KO efficiency^43, 59–62^ (Fig. 2). In a recent study, the improvement in KO efficiency with the use of three sgRNAs (triple-CRISPR) was examined in depth. Based on simulation, the average KO efficiency expected with single sgRNA was around 60% and triple-CRISPR would increase the rate to over 80%. The actual rate reached to over 95% due to long deletions between CRISPR targeted sites.^62^ A set of triple sgRNAs which cover ~80% of all genes in the mouse genome has been created as an open database (http://crispr.riken.jp/). Potential off-target effects can be also excluded by using the second set of triple sgRNAs that covers ~70% of all mouse genes.^62^

### Introduction of DNA fragments

Targeted insertion or KI of a DNA fragment with mutated sequence, short functional sequence (restriction enzyme site, recombinase recognition site, or protein tag etc.), or functional expression cassette can be also facilitated via HDR, NHEJ and MMEJ by co-transfer of linear or circular donor vector, PCR fragment or single-stranded oligo DNA nucleotide (ssODN) together with the site-specific endonucleases (Fig. 2).

Homologous recombination (HR)-dependent targeting is mediated by a form of HDR. This pathway has been widely used for a large fragment insertion or KI both in cultured cells and zygotes by using a donor targeting vector with long homology arms.^43, 44, 63–69^ The targeting rate is relatively low but efficiently enriched by antibiotic drug selection in the culture. A shorter functional sequence or small mutation can be more simply introduced by using ssODN.^51, 55, 59, 66, 70–75^

NHEJ-mediated fragment insertion/KI is easier and more efficient than the HR pathway, because the NHEJ-repair reaction is thought to predominate over the HR reaction for DSB repair.^76, 77^ In the NHEJ-mediated insertion, both the donor plasmid and the target genome loci are digested simultaneously. And then, the digested donor plasmid is integrated into the digested genome loci. A PCR fragment or double-stranded ODN can be also applied as an integrated fragment without digestion. This pathway works not only in cultured mammalian cells (including ESCs) but also in zebrafish, and does not necessarily require antibiotic selection.^56, 78–81^ In addition, there is no need to prepare a targeting vector with long homology arms, which is generally a time-consuming process. On the other hand, it is of note that the direction of the inserted fragment is not controllable, and indels are usually introduced at the junction site. Therefore, the method is inappropriate for some KI purposes, such as in-frame KI of an exogenous ORF into an endogenous gene.

MMEJ-mediated editing provides more simplified KI strategy with precise direction and junction sequence. Instead of the conventionally used long homology arms for HR-mediated KI, this pathway uses only extremely short microhomology sequences (10–40 bp) for the precise fragment insertion. MMEJ-mediated KI also works in mammalian cells, and the inserted fragment can be supplied as an in vivo digested plasmid or a PCR fragment.^82, 83^ Therefore, the editing pathway potentially overcomes problems in HR-mediated or NHEJ-mediated KI.

## Current technologies for one-step production of genome-edited mice

### Direct genome editing in one-cell zygotes

The compelling advantages of the site-specific endonucleases in efficient genome-editing has been examined in recent years. In particular, zygotic genome editing enables one-step production of genome-edited animals, skipping the in vitro targeting step in ESCs. Introduction of components into one-cell zygotes are relatively simple and easy, particularly for the CRISPR/Cas9 system, which just requires cytoplasmic microinjection or electroporation.^84–87^

**Fig. 3:**
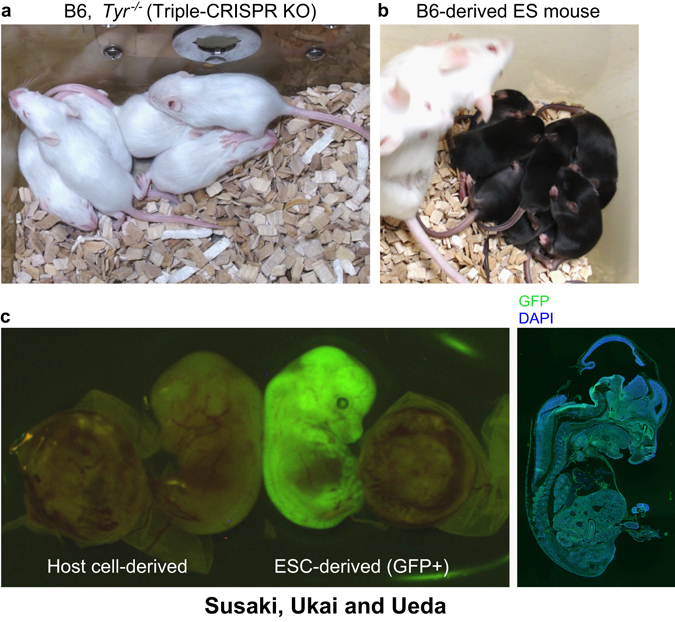
One-step generation of genome-edited mice. **a** An example of triple-CRISPR KO littermates (before weaning) in B6 strain. *Tyr* gene coding tyrosinase (an enzyme involving black coat color) was knocked out by the triple-CRISPR method.^62^ All littermates had white coat color, indicating ~100% bi-allelic KO rate of the targeted gene. **b** An example of B6 ES mouse littermates (before weaning) by 3i + LIF culture and 8-cell injection.^141^ All littermates had black coat color, indicating efficient generation of ~100% ESC-derived mouse. **c** An ES mouse embryo (E13.5) derived from an H2B-EGFP KI ESC clone.^170^ Only the embryo (but not the extraembryonic tissues) expresses EGFP, suggesting the unique contribution of ESC-derived cells. Entire section of the ES mouse is shown in the right panel. All animal experiments here were approved by the Institutional Animal Care and Use Committee of RIKEN Kobe Branch, and all of the animal care was in accordance with the Institutional Guidelines

KO animals can be generated in a one-step manner by exploiting endonuclease-mediated DSBs followed by NHEJ with indel insertion in zygotes. In an earlier study, ZFN was tested in rat zygotes,^57^ where up to 75% of live-born F0 founders were harboring mutations. TALEN has been similarly tested,^88^ while CRISPR/Cas9 was mainly used in the most recent studies, since 2013.^59, 60, 89, 90^ This method accelerates the generation of KO animals via the co-injection of RNA encoding the Cas9 protein and target-locus-specific guide RNAs into embryos. Long deletions of a genomic region (10–100 kb) were induced by using two sgRNAs.^60, 91, 92^ Others reported F0 phenotyping of CRISPR/Cas9 KO animals,^93^ suggesting the potential of this method for use in next-generation genetics schemes. Several modifications of the CRISPR/Cas9 system have been also introduced to improve the efficiency and specificity of targeted mutations in a genome.^51, 52, 55, 61^ However, two problems have remained: (1) first-generation mice often contain a mosaic of wild-type and KO cells, and (2) the rate of whole-body bi-allelic mutant mice generated is relatively low (usually ~60–80% at best). Therefore, the highly efficient (>90%) production of whole-body bi-allelic KO in a single generation remained a fundamental challenge for next-generation mammalian genetics. To realize this vision of next-generation mammalian genetics, the triple-CRISPR method significantly improved bi-allelic modification efficiency and further elicited almost perfect (~100%) whole-body bi-allelic KO mice^62^ (Fig. 3a). It is of note that this was performed with B6 zygotes so that the resulted KO animals could be used for the subsequent experiments without backcross. Taken together, next-generation mammalian genetics has been achieved, at least for the production of KO mice (Fig. 1b).

On the other hand, one-step production of KI mice (zygotic KI) is still under development. An earlier study using ZFN reported that the KI mice were generated in one-step manner at the production rate of 1.7–4.5% (% of KI pups/all pups).^65^ This was considered outstanding given that a spontaneous recombination rate is ~0.1% in zygotes.^94^ In more recent studies, introducing mutations (including multiplexed editing), short functional sequences or even a large reporter cassette were tested mainly by using CRISPR/Cas9 system.^59–61, 66, 85, 91, 92, 95–101^ In contrast to the improved KO rates in the one-step production scheme, zygotic KI by HDR still remains inefficient, particularly in the case of long fragment insertion by homologous recombination (initially ~20%^84^). Several studies have tried to improve the genome-editing (KI) rate. For example, inhibition of the NHEJ pathway by administration of DNA ligase IV inhibitor (Scr7) gives a 2 to 4-fold increase of HDR rate in mouse zygotes,^97^ although another study debated the capacity of this inhibitor in human models.^102^ Similarly, the treatment with an actin polymerization inhibitor (cytochalasin B or D) increases the HDR targeting rate presumably due to the delayed DSB repair.^99^ The use of Cas9 protein rather than synthesized mRNA also increases HDR rate.^92, 96, 98^ One of these studies showed an increase of the genome-editing (KI) rate, up to ~45% KI efficiency of live-born pups by injecting Cas9 protein complex with synthesized dual-crRNA:tracrRNA into pronuclei.^98^ The use of Cas9 protein also reduces mosaicism when introduced with proper timing in early zygotes.^101^ Another study reported the generation of two KI newborns out of 123 injected embryos, where one was heterozygous KI while another was homozygous KI.^92^ ssODN-mediated KI, in which the cut sites of the targeted genome and the inserted fragment are ligated with 80-bp ssODNs homologous to the two cut ends (Fig. 2), was also shown to integrate up to ~200 kb of bacterial artificial chromosome (BAC) into the targeted locus.^100^ Finally, it was recently reported that HDR accuracy is dramatically increased by incorporating silent CRISPR/Cas-blocking mutations in sgRNA or Cas9-targeted sequence (CORRECT method)^103^ although testing this in zygotes still remains to be done. Please note however, that the reproducibility of these recent studies should be further examined because some of the conclusions are based on small number of experiments. Further improvements in the targeting rate and specificity will be needed for one-step generation of KI mice.

### ESC-derived mouse (ES mouse)

Although one-step generation of KI mice would be ideal for next-generation mammalian genetics, a number of issue have yet to be overcome, specifically inefficient editing and live-birth rate (particularly with a large fragment KI) and undesirable indel insertion and mosaicism in resultant animals. Alternatively, two-step generation of KI mice by almost completely ESC-derived mouse (“ES mouse”) is an attractive alternative at present. The advantages of using ESCs are in its selectivity of sex, easier storage and flexibility for more complex genome editing (e.g. multi-gene KO and KI) in in vitro culture. Furthermore, genome editing in ESCs is becoming easier by the site-specific nucleases.^65–67, 84, 91, 104, 105^ In recent studies, an HDR targeting rate over 10% was achieved in ESCs even using very short (0.5 kb) homologous arms together with CRISPR/Cas9.^104^ Multiple KO ESCs were also prepared in one-step manner, at the rate of approximately 20% in a triple KO experiment.^59^ The other modern genome-editing methods discussed above may potentially to be applicable as well.

Validity of ES mouse production and phenotyping analysis within a single generation was first proposed and tested by using the tetraploid complementation method.^106–113^ However, several possible drawbacks of the method are known. First, substantial contamination of host cells was often observed in chimera mice produced by this method, which can cause developmental abnormalities.^113–115^ Second, the genetic heterozygosity of both tetraploid embryos and ESCs seems to be crucial for survival of the resultant ES mice,^110, 112, 114^ which means that the use of inbred ESCs does not seem possible and further backcrossing is required. In addition, preparing hundreds of tetraploid embryos every time does not seem practical for routine generation of many ES mice. For these reasons, few reports have used tetraploid complementation in a large-scale phenotyping assay of ES mice.

Alternatively, ES mice can be generated by ESCs injected into or aggregated with eight-cell embryos rather than the conventionally used blastocyst embryos.^116–118^ The eight-cell injection/aggregation, in which totipotent host cells and ESCs as epiblast precursors are used, produces almost completely ESC-derived mice with ~0.1% contamination of host embryo cells. Furthermore, it is striking that ES mice from inbred strains, such as B6 and BALB/C were efficiently generated at the live-birth rates of 11~29% (ES mice/embryos transferred), which was comparable to the 129 strain (9~15%) and hybrid ES mice (6~40%) and significantly higher than in blastocyst injection in any case (all 0%).^117^ Therefore, F0 phenotyping of inbred ES mice generated with 8-cell injection/aggregation was considered plausible in the study. Possible drawbacks would be that the ES mouse production rate might depend on the ‘quality’ of cultured ESCs. A further optimized method to stably maintain ESCs in their naive pluripotent state was thus required.

Secretory regulatory factors and their downstream mechanisms for maintaining ESC’s naive pluripotency have been well studied (recently reviewed in Huang et al.^119^ and others). Historically, the leukemia inhibitory factor–signal transducer and activator of transcription 3 (LIF/Stat3) pathway has been found to be indispensable for maintaining pluripotency and self-renewal ability of ESC.^120–123^ More recently, additional pathways essential for ESC maintenance or differentiation were uncovered. One is the Wnt–β-catenin pathway which supports ESC propagation and maintenance of the pluripotent naive state, and is antagonized by glycogen synthase kinase-3 (Gsk3)–Tcf3 (also known as Tcf7l1).^124–128^ The other is fibroblast growth factor 4–mitogen-activated protein kinase (Mek)–mitogen-activated protein kinase (Mapk or Erk) pathway which leads ESC differentiation and thus its inhibition suppresses ESC differentiation.^129, 130^ Therefore, naive pluripotent ESCs can be stably maintained by shielding the cells from these differentiation triggers, and by addition of inhibitors (3i: SU5402 for FGF receptor, PD184352 for Mek, CHIR99021 for Gsk3, or 2i: PD0325901 for Mek and CHIR99021 for Gsk3) and in serum-free conditions.^131–133^ 3i/2i-cultured ESCs exhibit restricted expressions of lineage-affiliated genes and stabilized gene expressions involving a naive pluripotent state via epigenetic modulations and appropriate control of the pluripotency factors.^134–139^

The administration of 3i/2i enabled more efficient creation and maintenance of ESCs from even inbred mouse strains (including B6) or rat,^133, 140–142^ and increased germ-line transmission of B6-derived ESC chimera.^116^ The stable establishment and maintenance of B6 ESCs in ES mouse production is particularly critical for the next-generation mammalian genetics without crossing. Previously, B6 ESCs were suggested to have problems of maintenance, less efficient chimera formation and germ-line transmission, and genomic instability in standard culture conditions.^143, 144^ To overcome these problems, B6 ESCs were established and maintained in serum-free 3i/2i + LIF medium, which demonstrates significantly higher success rate (67 vs. 3% in media containing serum)^141^ (Fig. 3b, c). In addition, ~100% ESC-derived mice from the B6-3i ESCs can be stably generated with eight-cell injection, even after many passages and traditional homologous-recombined targeting, at a production rate of 30~100% (ES mice/live-born mice). Therefore, 3i/2i + LIF culture and eight-cell injection/aggregation of ESCs enables the efficient one-step generation of ES mice, and subsequent F0 phenotyping can be performed once the genome-edited ES mice are created. Indeed, production and data acquisition of a novel ES mouse strain expressing a bright fluorescent protein was completed within a few months.^145, 146^ CRISPR/Cas9-mediated knock-in in 2i + LIF-cultured ESCs followed by eight-cell injection have also been performed.^147^ These examples support the potential of ES mouse schemes. Note that ICR/CD-1 host embryos can be used for the ES mouse production and there is no need for maintaining a specific Tg colony for host embryos. Furthermore, experimental procedures (such as an operation for implantation) are similar to, or even lesser than conventional chimera mouse production.

To further improve the efficiency of ES mouse production, better culture methods and ESC quality control will be needed. Genomic instability, in particular, should be avoided during culture because chromosomal aneuploidy can cause embryonic death. Telomere extension seems important for maintaining normal karyotype of ESCs, and frequent activation of telomere maintenance factor Zscan4 restores and maintains the ESC’s potency in long-term culture.^148, 149^ Aneuploidy detection in cell culture populations is also important for ESC’s quality control. This can be performed by not only a conventional karyotyping but also by a droplet digital PCR-based screening.^150^ Furthermore, additional chemical treatments can possibly ameliorate ESC culture conditions. So far, a variety of chemicals including ROCK inhibitor,^151, 152^ PKC inhibitor,^153, 154^ ERK/p38 inhibitor,^155^ HDAC inhibitors [e.g., trichostatin A, sodium butylate or valproic acid^156–158^ or Vitamin C^159^] may potentially contribute to improved potency of ESCs. Therefore, applications of these chemicals and routine quality control may help accelerate next-generation genetics based on ES mouse production.

## Practice of next-generation mammalian genetics

As discussed above, high-throughput KO or KI mouse production is pivotal for accelerating system-level identification, and analysis of molecular networks and cellular circuits in organisms. Given that various genetic tools, such as optogenetics and chemogenetic tools^160, 161^ are developing rapidly in recent years, high-throughput genome-edited mouse production is required for their in vivo implementation. Next-generation mammalian genetics potentially enables a single laboratory or a single researcher to generate, maintain and analyze multiple genome-edited strains rather than institutes or consortiums for production, deposit and distribution of various strains and ESCs. Because sgRNAs for targeted sites can be readily designed and prepared, the CRISPR/Cas9 system, in particular, makes such large-scale genetics feasible. Indeed, a recent study generated 31 novel CRISPR-KO mice lacking testis-expressing genes.^162^ Since whole-body bi-allelic KO rates were not sufficiently high for the next-generation scheme, the authors performed the crossing of selected F0 founders based on sequence and PCR screening data. In order to realize almost perfect (~100%) whole-body bi-allelic KO rate for next-generation mammalian genetics, we recently performed triple-CRISPR-based large-scale reverse genetics for sleep research. To identify genes involving neural electrophysiological activities during sleep or wake, we first developed an average neuron model in silico and found that genes involved in intracellular Ca^2+^ regulation (Ca^2+^ channels, Ca^2+^-dependent channels, Ca^2+^-pumps or Ca^2+^-dependent enzymes) are important for electrophysiological slow-wave-oscillation patterns during sleep. To further assess the roles of these genes in vivo, we next produced KO mice for 33 genes with the triple-CRISPR methods and eventually identified 8 genes important for regulating sleep duration.^62, 163^

ES mouse technology can deal with more complex genome editing, which would be difficult, if not impossible, with the conventional crossing-based genetics. For example, we also produced ~20 KO-rescue ES mice in order to perform system-level analysis of circadian clock-gene circuits in organisms.^164^ In this experiment, a 3i + LIF-cultured ESC clone derived from a double-KO mouse lacking two core clock genes (*Cry1* and *Cry2*) was established. A rescue expression cassette of the wild-type or mutated *Cry1* gene was then homologously knocked-in in the ESC clone (thus five alleles had been edited). Double-KO ES mice and KO-rescue ES mice were then generated and used for F0 phenotyping to measure in vivo 24 h rhythmicity. As explicitly indicated by these examples, next-generation mammalian genetics enables large-scale organism-level experiments within reasonable time, space and labor.

Next-generation genetics is also important for improving animal welfare and 3R principles, particularly contributing to “reduction” of animal use. In our triple-CRISPR experiments,^62^ the yields of bi-allelic KO mice lacking tyrosinase gene (judged by the white coat color) were 17% on average, and 36% in the best case, of the injected and transferred B6 zygotes. Therefore, between 60 (average) and 30 (best case) of host embryos would be enough for generating a sufficient number (around 10) of bi-allelic KO mice. The rate of bi-allelic tyrosinase KO mice among the F0 littermates was 97.5% on average and 100% at best. Similarly, at least in our ES-mouse experiments of *Cry1* rescue in the *Cry1*/*Cry2* DKO background,^164^ the yield of ES mice available for phenotyping was 5.5% on average, and 35% in the best case, of the injected 8-cell embryos. Therefore, between 170 (average) and 30 (best case) of host embryos would be enough for generating a sufficient number (around 10) of ES mice. The rate of ES mice among the F0 littermates was 43% as average and 91% at the best. Only the littermates of embryonic lethal, non-KO or non-ES mice were sacrificed and no further animals are needed. The number of animals used is thus much smaller than the conventional methods, in which a similar number of host embryos are used for injection, and only a part of the founders or chimera mice are used for further crossing. In the conventional case, dozens of littermates are produced and sacrificed during crossing to select mice with an expected genotype. With conventional methods the number needed exponentially increases when a more complicated genetic background (e.g., double KO) is desired, while with next-generation genetics the number of used animals is not dependent on genetic complexity.

On the other hand, researchers need to take special care regarding some issues with the use of F0 animals for phenotype studies. In particular, researchers should carefully consider to what extent potential mosaicism (e.g., mutational variations in the triple-CRISPR method, or undetectable contamination of wild-type cells in the ES mouse method) would affect the final results of a scientific study. In our above experiments, the phenotypic variations of F0 mice were comparable with those in wild-type or suitable control animals,^62, 163, 164^ suggesting that mutational variations (triple-CRISPR) or undetectable contamination of wild-type cells (ES mouse) do not seem problematic at lease in these cases. To further exclude the possibility of artifact phenotypes due to mutational variations or undetectable contamination of wild-type cells, we recommend that researchers independently generate a whole-body bi-allelic KO mice by using a second set of triple-CRISPR for the same gene, or to independently generate a whole-body bi-allelic KI mice using an independent clone of ES cells. Such stringent criteria (the production of independent KO or KI mice to confirm the observed phenotype) is sometimes difficult to fulfill with conventional mouse genetics because it takes a couple of years for another round of production. On the other hand, this step is feasible with next-generation genetics because it only takes a couple of months. In this sense, the quality of scientific studies using next-generation genetics should exceed that of scientific studies using conventional genetics.

Another point that researchers need to consider carefully is experimental design. This includes (1) preparation of appropriate control animals without any obvious defects in the focused phenotype (e.g., ES mice from wild-type ESCs or ESCs without genome editing for a clock gene rescue study,^164^ and tyrosinase triple-CRISPR KO mice for a sleep study^62^), (2) evaluation of genetic composition of the F0 animals with strict criteria (e.g., detection of a genomic deletion as a proof of efficient triple-CRISPR method^62, 163^ or a highly sensitive detection of contaminated host embryo-derived cells^164^). In addition, it is also useful, if necessary, to evaluate undesirable mutations in the coding sequences by exon sequencing^163^ of triple-CRISPR KO mice. It is also useful to avoid cumulative mutations^165^ by using ESCs with minimal passage numbers for the production of ES mice. Moreover, these experimental procedures should be described according to a general guideline for animal experiments (e.g., the ARRIVE guidelines^166^). Further efforts to minimize problems from these issues should be expanded in future studies.

## Perspectives

Next-generation mammalian genetics will facilitate system-level analysis of molecular and cellular circuits in organisms. To further improve the throughput for genome-edited animal production, additional developments of new technologies related to next-generation mammalian genetics will be required. For example, one-step production of whole-body bi-allelic KI mice by more efficient genome editing of zygotes still remains unachieved. For the two-step production of KI mice using ES mouse technology, the preparation of host embryos and surrogate mothers are still labor intensive and pose limitations on its throughput. New technologies which overcome these limitations will further accelerate next-generation mammalian genetics and also reduce the number of experimental animals used to obtain the same information.

Next-generation mammalian genetics together with efficient, quantitative and non-invasive phenotyping methods will provide an attractive platform for investigating the organism-level functions of the molecular and cellular circuits of interests. Genome-wide phenome analysis has been performed in international KO mouse phenotyping efforts^18, 167^ to systematically survey the functions of molecular networks in organisms. Although most large-scale organism-level phenotyping projects are usually labor-intensive, the development of more facile alternatives is possible. Recently, whole-body clearing and imaging methods with single-cell resolution have been developed^146, 168, 169^ and started to provide comprehensive and quantitative experimental data at the cell-to-organism level, further facilitating organism-level systems biology. The development of non-invasive phenotyping will be also an attractive direction. For example, sleep phenotyping represents such a recent attempt where a non-invasive sleep phenotyping system was used instead of a conventional invasive EEG/EMG-based measurement system. In fact, these non-invasive methods have already enabled sleep phenotyping of dozens of triple-CRISPR KO mice.^62, 163^ Organism-level systems biology is thus coming to fruition with next-generation mammalian genetics, whole-body clearing and imaging with single-cell resolution, and non-invasive and quantitative phenotyping methods. Organism-level systems biology based on such new technologies will accelerate our understanding of complex and dynamic molecular and cellular circuits in the near future.

## References

[1] Kitano H (2002). Systems biology: a brief overview. Science.

[2] Kitano H (2002). Computational systems biology. Nature.

[3] Ukai H, Ueda HR (2010). Systems biology of mammalian circadian clocks. Annu. Rev. Physiol..

[4] Susaki EA, Ueda HR (2016). Whole-body and whole-organ clearing and imaging techniques with single-cell resolution: toward organism-level systems biology in mammals. Cell Chem. Biol..

[5] Evans MJ, Kaufman MH (1981). Establishment in culture of pluripotential cells from mouse embryos. Nature.

[6] Martin GR (1981). Isolation of a pluripotent cell line from early mouse embryos cultured in medium conditioned by teratocarcinoma stem cells. Proc. Natl. Acad. Sci. USA.

[7] Capecchi MR (2005). Gene targeting in mice: functional analysis of the mammalian genome for the twenty-first century. Nat. Rev. Genet..

[8] Jaenisch R (1988). Transgenic animals. Science.

[9] Gerlai R (1996). Gene-targeting studies of mammalian behavior: is it the mutation or the background genotype?. Trends Neurosci..

[10] Vitaterna MH (1994). Mutagenesis and mapping of a mouse gene, clock, essential for circadian behavior. Science.

[11] Takahashi JS, Pinto LH, Vitaterna MH (1994). Forward and reverse genetic approaches to behavior in the mouse. Science.

[12] King DP (1997). Positional cloning of the mouse circadian clock gene. Cell.

[13] Brown SD, Nolan PM (1998). Mouse mutagenesis-systematic studies of mammalian gene function. Hum. Mol. Genet..

[14] Nolan PM (2000). A systematic, genome-wide, phenotype-driven mutagenesis programme for gene function studies in the mouse. Nat. Genet..

[15] Guan C, Ye C, Yang X, Gao J (2010). A review of current large-scale mouse knockout efforts. Genesis.

[16] Collins FS, Rossant J, Wurst W (2007). A mouse for all reasons. Cell.

[17] Skarnes WC (2011). A conditional knockout resource for the genome-wide study of mouse gene function. Nature.

[18] White JK (2013). Genome-wide generation and systematic phenotyping of knockout mice reveals new roles for many genes. Cell.

[19] Dickinson ME (2016). High-throughput discovery of novel developmental phenotypes. Nature.

[20] Murray SA, Eppig JT, Smedley D, Simpson EM, Rosenthal N (2012). Beyond knockouts: cre resources for conditional mutagenesis. Mamm. Genome.

[21] Gerfen CR, Paletzki R, Heintz N (2013). GENSAT BAC cre-recombinase driver lines to study the functional organization of cerebral cortical and basal ganglia circuits. Neuron.

[22] Taniguchi H (2011). A resource of Cre driver lines for genetic targeting of GABAergic neurons in cerebral cortex. Neuron.

[23] Morrical SW (2015). DNA-pairing and annealing processes in homologous recombination and homology-directed repair. Cold Spring Harb. Perspect. Biol..

[24] Lieber MR (2010). The mechanism of double-strand DNA break repair by the nonhomologous DNA end-joining pathway. Annu. Rev. Biochem..

[25] Decottignies A (2013). Alternative end-joining mechanisms: a historical perspective. Front. Genet.

[26] Rouet P, Smih F, Jasin M (1994). Expression of a site-specific endonuclease stimulates homologous recombination in mammalian cells. Proc. Natl. Acad. Sci. USA.

[27] Chandrasegaran S, Carroll D (2016). Origins of programmable nucleases for genome engineering. J. Mol. Biol..

[28] Urnov FD, Rebar EJ, Holmes MC, Zhang HS, Gregory PD (2010). Genome editing with engineered zinc finger nucleases. Nat. Rev. Genet..

[29] Sommer D, Peters AE, Baumgart AK, Beyer M (2015). TALEN-mediated genome engineering to generate targeted mice. Chromosome Res..

[30] Sander JD, Joung JK (2014). CRISPR-Cas systems for editing, regulating and targeting genomes. Nat. Biotechnol..

[31] Vanamee ES, Santagata S, Aggarwal AK (2001). FokI requires two specific DNA sites for cleavage. J. Mol. Biol..

[32] Hale CR (2009). RNA-guided RNA cleavage by a CRISPR RNA-Cas protein complex. Cell.

[33] Miller J, McLachlan AD, Klug A (1985). Repetitive zinc-binding domains in the protein transcription factor IIIA from Xenopus oocytes. EMBO J..

[34] Beerli RR, Barbas CF (2002). Engineering polydactyl zinc-finger transcription factors. Nat. Biotechnol..

[35] Wolfe SA, Nekludova L, Pabo CO (2000). DNA recognition by Cys2His2 zinc finger proteins. Annu. Rev. Biophys. Biomol. Struct..

[36] Maeder ML (2008). Rapid “open-source” engineering of customized zinc-finger nucleases for highly efficient gene modification. Mol. Cell.

[37] Sander JD (2011). Selection-free zinc-finger-nuclease engineering by context-dependent assembly (CoDA). Nat. Methods.

[38] Gupta A (2012). An optimized two-finger archive for ZFN-mediated gene targeting. Nat. Methods.

[39] Moscou MJ, Bogdanove AJ (2009). A simple cipher governs DNA recognition by TAL effectors. Science.

[40] Boch J (2009). Breaking the code of DNA binding specificity of TAL-type III effectors. Science.

[41] Kim Y (2013). A library of TAL effector nucleases spanning the human genome. Nat. Biotechnol..

[42] Jinek M (2012). A programmable dual-RNA-guided DNA endonuclease in adaptive bacterial immunity. Science.

[43] Cong L (2013). Multiplex genome engineering using CRISPR/Cas systems. Science.

[44] Mali P (2013). RNA-guided human genome engineering via Cas9. Science.

[45] Wang T, Wei JJ, Sabatini DM, Lander ES (2014). Genetic screens in human cells using the CRISPR-Cas9 system. Science.

[46] Shalem O (2014). Genome-scale CRISPR-Cas9 knockout screening in human cells. Science.

[47] Mojica FJ, Diez-Villasenor C, Garcia-Martinez J, Almendros C (2009). Short motif sequences determine the targets of the prokaryotic CRISPR defence system. Microbiology.

[48] Kleinstiver BP (2015). Engineered CRISPR-Cas9 nucleases with altered PAM specificities. Nature.

[49] Hirano S, Nishimasu H, Ishitani R, Nureki O (2016). Structural basis for the altered PAM specificities of engineered CRISPR-Cas9. Mol. Cell.

[50] Sapranauskas R (2011). The streptococcus thermophilus CRISPR/Cas system provides immunity in Escherichia coli. Nucleic Acids Res..

[51] Ran FA (2013). Double nicking by RNA-guided CRISPR Cas9 for enhanced genome editing specificity. Cell.

[52] Shen B (2014). Efficient genome modification by CRISPR-Cas9 nickase with minimal off-target effects. Nat. Methods.

[53] Tsai SQ (2014). Dimeric CRISPR RNA-guided FokI nucleases for highly specific genome editing. Nat. Biotechnol..

[54] Guilinger JP, Thompson DB, Liu DR (2014). Fusion of catalytically inactive Cas9 to FokI nuclease improves the specificity of genome modification. Nat. Biotechnol..

[55] Fu Y, Sander JD, Reyon D, Cascio VM, Joung JK (2014). Improving CRISPR-Cas nuclease specificity using truncated guide RNAs. Nat. Biotechnol..

[56] Gaj T, Gersbach CA, Barbas CF (2013). ZFN, TALEN, and CRISPR/Cas-based methods for genome engineering. Trends Biotechnol..

[57] Geurts AM (2009). Knockout rats via embryo microinjection of zinc-finger nucleases. Science.

[58] Sung YH (2013). Knockout mice created by TALEN-mediated gene targeting. Nat. Biotechnol..

[59] Wang H (2013). One-step generation of mice carrying mutations in multiple genes by CRISPR/Cas-mediated genome engineering. Cell.

[60] Fujii W, Kawasaki K, Sugiura K, Naito K (2013). Efficient generation of large-scale genome-modified mice using gRNA and CAS9 endonuclease. Nucleic Acids Res..

[61] Zhou J (2014). Dual sgRNAs facilitate CRISPR/Cas9-mediated mouse genome targeting. FEBS J..

[62] Sunagawa GA (2016). Mammalian reverse genetics without crossing reveals Nr3a as a short-sleeper gene. Cell Rep.

[63] Hockemeyer D (2009). Efficient targeting of expressed and silent genes in human ESCs and iPSCs using zinc-finger nucleases. Nat. Biotechnol..

[64] Hockemeyer D (2011). Genetic engineering of human pluripotent cells using TALE nucleases. Nat. Biotechnol..

[65] Meyer M, de Angelis MH, Wurst W, Kuhn R (2010). Gene targeting by homologous recombination in mouse zygotes mediated by zinc-finger nucleases. Proc. Natl. Acad. Sci. USA.

[66] Yang H (2013). One-step generation of mice carrying reporter and conditional alleles by CRISPR/Cas-mediated genome engineering. Cell.

[67] Sommer D (2014). Efficient genome engineering by targeted homologous recombination in mouse embryos using transcription activator-like effector nucleases. Nat. Commun..

[68] Jones JM, Meisler MH (2014). Modeling human epilepsy by TALEN targeting of mouse sodium channel Scn8a. Genesis.

[69] Cui X (2011). Targeted integration in rat and mouse embryos with zinc-finger nucleases. Nat. Biotechnol..

[70] Wefers B (2013). Direct production of mouse disease models by embryo microinjection of TALENs and oligodeoxynucleotides. Proc. Natl. Acad. Sci. USA.

[71] Chen F (2011). High-frequency genome editing using ssDNA oligonucleotides with zinc-finger nucleases. Nat. Methods.

[72] Panda SK (2013). Highly efficient targeted mutagenesis in mice using TALENs. Genetics.

[73] Soldner F (2011). Generation of isogenic pluripotent stem cells differing exclusively at two early onset Parkinson point mutations. Cell.

[74] Shen B (2013). Efficient knockin mouse generation by ssDNA oligonucleotides and zinc-finger nuclease assisted homologous recombination in zygotes. PLoS One.

[75] Wang X (2014). Precise gene modification mediated by TALEN and single-stranded oligodeoxynucleotides in human cells. PLoS One.

[76] Sonoda E, Hochegger H, Saberi A, Taniguchi Y, Takeda S (2006). Differential usage of non-homologous end-joining and homologous recombination in double strand break repair. DNA Repair (Amst.).

[77] Shrivastav M, De Haro LP, Nickoloff JA (2008). Regulation of DNA double-strand break repair pathway choice. Cell Res..

[78] He X (2016). Knock-in of large reporter genes in human cells via CRISPR/Cas9-induced homology-dependent and independent DNA repair. Nucleic Acids Res..

[79] Geisinger JM, Turan S, Hernandez S, Spector LP, Calos MP (2016). In vivo blunt-end cloning through CRISPR/Cas9-facilitated non-homologous end-joining. Nucleic Acids Res..

[80] Auer TO, Duroure K, De Cian A, Concordet JP, Del Bene F (2014). Highly efficient CRISPR/Cas9-mediated knock-in in zebrafish by homology-independent DNA repair. Genome Res..

[81] Kimura Y, Hisano Y, Kawahara A, Higashijima S (2014). Efficient generation of knock-in transgenic zebrafish carrying reporter/driver genes by CRISPR/Cas9-mediated genome engineering. Sci. Rep..

[82] Nakade S (2014). Microhomology-mediated end-joining-dependent integration of donor DNA in cells and animals using TALENs and CRISPR/Cas9. Nat. Commun..

[83] Sakuma T (2015). Homologous recombination-independent large gene cassette knock-in in CHO cells using TALEN and MMEJ-Directed donor plasmids. Int. J. Mol. Sci..

[84] Yang H, Wang H, Jaenisch R (2014). Generating genetically modified mice using CRISPR/Cas-mediated genome engineering. Nat. Protoc..

[85] Horii T (2014). Validation of microinjection methods for generating knockout mice by CRISPR/Cas-mediated genome engineering. Sci. Rep..

[86] Kaneko T, Mashimo T (2015). Simple genome editing of rodent intact embryos by electroporation. PLoS One.

[87] Hashimoto M, Takemoto T (2015). Electroporation enables the efficient mRNA delivery into the mouse zygotes and facilitates CRISPR/Cas9-based genome editing. Sci. Rep.

[88] Tesson L (2011). Knockout rats generated by embryo microinjection of TALENs. Nat. Biotechnol..

[89] Shen B (2013). Generation of gene-modified mice via Cas9/RNA-mediated gene targeting. Cell Res..

[90] Mashiko D (2013). Generation of mutant mice by pronuclear injection of circular plasmid expressing Cas9 and single guided RNA. Sci. Rep.

[91] Zhang L (2015). Large genomic fragment deletions and insertions in mouse using CRISPR/Cas9. PLoS One.

[92] Wang L (2015). Large genomic fragment deletion and functional gene cassette knock-in via Cas9 protein mediated genome editing in one-cell rodent embryos. Sci. Rep.

[93] Sung YH (2014). Highly efficient gene knockout in mice and zebrafish with RNA-guided endonucleases. Genome Res..

[94] Brinster RL (1989). Targeted correction of a major histocompatibility class II E alpha gene by DNA microinjected into mouse eggs. Proc. Natl. Acad. Sci. USA.

[95] Inui M (2014). Rapid generation of mouse models with defined point mutations by the CRISPR/Cas9 system. Sci. Rep..

[96] Menoret S (2015). Homology-directed repair in rodent zygotes using Cas9 and TALEN engineered proteins. Sci. Rep..

[97] Maruyama T (2015). Increasing the efficiency of precise genome editing with CRISPR-Cas9 by inhibition of nonhomologous end joining. Nat. Biotechnol..

[98] Aida T (2015). Cloning-free CRISPR/Cas system facilitates functional cassette knock-in in mice. Genome Biol..

[99] Nakao H (2016). A possible aid in targeted insertion of large DNA elements by CRISPR/Cas in mouse zygotes. Genesis.

[100] Yoshimi K (2016). ssODN-mediated knock-in with CRISPR-Cas for large genomic regions in zygotes. Nat. Commun..

[101] Hashimoto M, Yamashita Y, Takemoto T (2016). Electroporation of Cas9 protein/sgRNA into early pronuclear zygotes generates non-mosaic mutants in the mouse. Dev. Biol..

[102] Greco GE (2016). SCR7 is neither a selective nor a potent inhibitor of human DNA ligase IV. DNA Repair (Amst.).

[103] Paquet D (2016). Efficient introduction of specific homozygous and heterozygous mutations using CRISPR/Cas9. Nature.

[104] Oji A (2016). CRISPR/Cas9 mediated genome editing in ES cells and its application for chimeric analysis in mice. Sci. Rep..

[105] Yu C (2015). Small molecules enhance CRISPR genome editing in pluripotent stem cells. Cell Stem Cell.

[106] Nagy A (1990). Embryonic stem cells alone are able to support fetal development in the mouse. Development.

[107] Nagy A, Rossant J, Nagy R, Abramow-Newerly W, Roder JC (1993). Derivation of completely cell culture-derived mice from early-passage embryonic stem cells. Proc. Natl. Acad. Sci. USA.

[108] Wang ZQ, Kiefer F, Urbanek P, Wagner EF (1997). Generation of completely embryonic stem cell-derived mutant mice using tetraploid blastocyst injection. Mech. Dev..

[109] Schwenk F (2003). Hybrid embryonic stem cell-derived tetraploid mice show apparently normal morphological, physiological, and neurological characteristics. Mol. Cell Biol..

[110] George SH (2007). Developmental and adult phenotyping directly from mutant embryonic stem cells. Proc. Natl. Acad. Sci. USA.

[111] Seibler J (2003). Rapid generation of inducible mouse mutants. Nucleic Acids Res..

[112] Li X (2005). The genetic heterozygosity and fitness of tetraploid embryos and embryonic stem cells are crucial parameters influencing survival of mice derived from embryonic stem cells by tetraploid embryo aggregation. Reproduction.

[113] Eakin GS, Hadjantonakis AK, Papaioannou VE, Behringer RR (2005). Developmental potential and behavior of tetraploid cells in the mouse embryo. Dev. Biol..

[114] Eggan K (2001). Hybrid vigor, fetal overgrowth, and viability of mice derived by nuclear cloning and tetraploid embryo complementation. Proc. Natl. Acad. Sci. USA.

[115] Lu TY, Markert CL (1980). Manufacture of diploid/tetraploid chimeric mice. Proc. Natl. Acad. Sci. USA.

[116] Gertsenstein M (2010). Efficient generation of germ line transmitting chimeras from C57BL/6N ES cells by aggregation with outbred host embryos. PLoS One.

[117] Poueymirou WT (2007). F0 generation mice fully derived from gene-targeted embryonic stem cells allowing immediate phenotypic analyses. Nat. Biotechnol..

[118] Huang J (2008). Efficient production of mice from embryonic stem cells injected into four- or eight-cell embryos by piezo micromanipulation. Stem Cells.

[119] Huang G, Ye S, Zhou X, Liu D, Ying QL (2015). Molecular basis of embryonic stem cell self-renewal: from signaling pathways to pluripotency network. Cell. Mol. Life Sci..

[120] Smith AG (1988). Inhibition of pluripotential embryonic stem cell differentiation by purified polypeptides. Nature.

[121] Williams RL (1988). Myeloid leukaemia inhibitory factor maintains the developmental potential of embryonic stem cells. Nature.

[122] Matsuda T (1999). STAT3 activation is sufficient to maintain an undifferentiated state of mouse embryonic stem cells. EMBO J..

[123] Niwa H, Burdon T, Chambers I, Smith A (1998). Self-renewal of pluripotent embryonic stem cells is mediated via activation of STAT3. Genes Dev..

[124] Wray J (2011). Inhibition of glycogen synthase kinase-3 alleviates Tcf3 repression of the pluripotency network and increases embryonic stem cell resistance to differentiation. Nat. Cell Biol..

[125] ten Berge D (2011). Embryonic stem cells require Wnt proteins to prevent differentiation to epiblast stem cells. Nat. Cell Biol..

[126] Sato N, Meijer L, Skaltsounis L, Greengard P, Brivanlou AH (2004). Maintenance of pluripotency in human and mouse embryonic stem cells through activation of Wnt signaling by a pharmacological GSK-3-specific inhibitor. Nat. Med..

[127] Pereira L, Yi F, Merrill BJ (2006). Repression of Nanog gene transcription by Tcf3 limits embryonic stem cell self-renewal. Mol. Cell Biol..

[128] Kielman MF (2002). Apc modulates embryonic stem-cell differentiation by controlling the dosage of beta-catenin signaling. Nat. Genet..

[129] Burdon T, Stracey C, Chambers I, Nichols J, Smith A (1999). Suppression of SHP-2 and ERK signalling promotes self-renewal of mouse embryonic stem cells. Dev. Biol..

[130] Kunath T (2007). FGF stimulation of the Erk1/2 signalling cascade triggers transition of pluripotent embryonic stem cells from self-renewal to lineage commitment. Development.

[131] Ying QL, Stavridis M, Griffiths D, Li M, Smith A (2003). Conversion of embryonic stem cells into neuroectodermal precursors in adherent monoculture. Nat. Biotechnol..

[132] Ying QL (2008). The ground state of embryonic stem cell self-renewal. Nature.

[133] Buehr M (2008). Capture of authentic embryonic stem cells from rat blastocysts. Cell.

[134] Singer ZS (2014). Dynamic heterogeneity and DNA methylation in embryonic stem cells. Mol. Cell.

[135] Marks H (2012). The transcriptional and epigenomic foundations of ground state pluripotency. Cell.

[136] Dunn SJ, Martello G, Yordanov B, Emmott S, Smith AG (2014). Defining an essential transcription factor program for naive pluripotency. Science.

[137] Ficz G (2013). FGF signaling inhibition in ESCs drives rapid genome-wide demethylation to the epigenetic ground state of pluripotency. Cell Stem Cell.

[138] Habibi E (2013). Whole-genome bisulfite sequencing of two distinct interconvertible DNA methylomes of mouse embryonic stem cells. Cell Stem Cell.

[139] Leitch HG (2013). Naive pluripotency is associated with global DNA hypomethylation. Nat. Struct. Mol. Biol..

[140] Batlle-Morera L, Smith A, Nichols J (2008). Parameters influencing derivation of embryonic stem cells from murine embryos. Genesis.

[141] Kiyonari H, Kaneko M, Abe S, Aizawa S (2010). Three inhibitors of FGF receptor, ERK, and GSK3 establishes germline-competent embryonic stem cells of C57BL/6N mouse strain with high efficiency and stability. Genesis.

[142] Li P (2008). Germline competent embryonic stem cells derived from rat blastocysts. Cell.

[143] Hughes ED (2007). Genetic variation in C57BL/6 ES cell lines and genetic instability in the Bruce4 C57BL/6 ES cell line. Mamm. Genome.

[144] Seong E, Saunders TL, Stewart CL, Burmeister M (2004). To knockout in 129 or in C57BL/6: that is the question. Trends Genet..

[145] Susaki EA (2014). Whole-brain imaging with single-cell resolution using chemical cocktails and computational analysis. Cell.

[146] Tainaka K (2014). Whole-body imaging with single-cell resolution by tissue decolorization. Cell.

[147] Wang Y (2016). Highly efficient generation of biallelic reporter gene knock-in mice via CRISPR-mediated genome editing of ESCs. Protein Cell.

[148] Amano T (2013). Zscan4 restores the developmental potency of embryonic stem cells. Nat. Commun..

[149] Zalzman M (2010). Zscan4 regulates telomere elongation and genomic stability in ES cells. Nature.

[150] Codner GF (2016). Aneuploidy screening of embryonic stem cell clones by metaphase karyotyping and droplet digital polymerase chain reaction. BMC Cell Biol..

[151] Watanabe K (2007). A ROCK inhibitor permits survival of dissociated human embryonic stem cells. Nat. Biotechnol..

[152] Zhang P, Wu X, Hu C, Wang P, Li X (2012). Rho kinase inhibitor Y-27632 and accutase dramatically increase mouse embryonic stem cell derivation. In Vitro Cell. Dev. Biol. Anim..

[153] Takashima Y (2014). Resetting transcription factor control circuitry toward ground-state pluripotency in human. Cell.

[154] Dutta D (2011). Self-renewal versus lineage commitment of embryonic stem cells: protein kinase C signaling shifts the balance. Stem Cells.

[155] Qi X (2004). BMP4 supports self-renewal of embryonic stem cells by inhibiting mitogen-activated protein kinase pathways. Proc. Natl. Acad. Sci. USA.

[156] Lee JH, Hart SR, Skalnik DG (2004). Histone deacetylase activity is required for embryonic stem cell differentiation. Genesis.

[157] Ware CB (2009). Histone deacetylase inhibition elicits an evolutionarily conserved self-renewal program in embryonic stem cells. Cell Stem Cell.

[158] Hezroni H, Sailaja BS, Meshorer E (2011). Pluripotency-related, valproic acid (VPA)-induced genome-wide histone H3 lysine 9 (H3K9) acetylation patterns in embryonic stem cells. J. Biol. Chem..

[159] Blaschke K (2013). Vitamin C induces Tet-dependent DNA demethylation and a blastocyst-like state in ES cells. Nature.

[160] Roth BL (2016). DREADDs for Neuroscientists. Neuron.

[161] Fenno L, Yizhar O, Deisseroth K (2011). The development and application of optogenetics. Annu. Rev. Neurosci..

[162] Miyata H (2016). Genome engineering uncovers 54 evolutionarily conserved and testis-enriched genes that are not required for male fertility in mice. Proc. Natl. Acad. Sci. USA.

[163] Tatsuki F (2016). Involvement of Ca(2+)-Dependent Hyperpolarization in sleep duration in mammals. Neuron.

[164] Ode KL (2017). Knockout-rescue embryonic stem cell-derived mouse reveals circadian-period control by quality and quantity of CRY1. Mol. Cell.

[165] Vanden Berghe T (2015). Passenger mutations confound interpretation of all genetically modified congenic mice. Immunity.

[166] Kilkenny C, Browne WJ, Cuthill IC, Emerson M, Altman DG (2010). Improving bioscience research reporting: the ARRIVE guidelines for reporting animal research. PLoS Biol..

[167] Brown SD, Moore MW (2012). Towards an encyclopaedia of mammalian gene function: the International mouse phenotyping consortium. Dis. Model Mech..

[168] Yang B (2014). Single-cell phenotyping within transparent intact tissue through whole-body clearing. Cell.

[169] Pan C (2016). Shrinkage-mediated imaging of entire organs and organisms using uDISCO. Nat. Methods.

[170] Abe T (2011). Establishment of conditional reporter mouse lines at ROSA26 locus for live cell imaging. Genesis.

